# Characterization of Reproductive and Morphological Variables in Female Elite Futsal Players

**DOI:** 10.3389/fpsyg.2021.625354

**Published:** 2021-05-05

**Authors:** Marcos Roberto Queiroga, Danilo Fernandes da Silva, Sandra Aires Ferreira, Vinícius Müller Reis Weber, Daniel Zanardini Fernandes, Timothy Gustavo Cavazzotto, Bruno Sergio Portela, Marcus Peikriszwili Tartaruga, Matheus Amarante Nascimento, Edgar Ramos Vieira

**Affiliations:** ^1^Department of Physical Education, Midwestern Paraná State University – UNICENTRO, Guarapuava, Brazil; ^2^Faculty of Health Sciences, School of Human Kinetics, University of Ottawa, Ottawa, ON, Canada; ^3^Associated Graduate Program in Physical Education UEM/UEL, Londrina, Brazil; ^4^Department of Physical Education, Paraná State University – UNESPAR, Paranavaí, Brazil; ^5^Department of Physical Therapy, Florida International University, Miami, FL, United States

**Keywords:** female athletes, sports medicine, early puberty, five-a-side football, reproductive health, menstrual cycle, soccer, women

## Abstract

We aimed to characterize the age of onset of training, age at menarche, menstrual periodicity, and performance perception during the menstrual cycle and examined the impact of these reproductive variables on body composition, morphology, and body weight satisfaction in Brazilian elite futsal players. The study consisted of 115 female Brazilian elite futsal players from the top national teams. Data were collected during the twentieth Women’s Brazil Futsal Cup. Players were interviewed and self-reported their age of onset of training, age at menarche, menstrual periodicity, and the menstrual period, where they performed best. We also asked for what they considered to be their ideal body weight as well as information related to their training (i.e., volume and frequency). Subsequently, anthropometric measurements (i.e., body mass, height, circumferences, diameters, and skinfold thickness) were performed to estimate the body composition and determine morphological characteristics (e.g., somatotype). Fifty-nine (53.2%) players were postmenarche-trained and 52 (46.8%) were premenarche-trained. Eighteen (16.2%), 65 (58.6%), and 28 (25.2%) were classified as early, normal, and late menarche, respectively. Only 11 (9.6%) and 1 (0.9%) had irregular menstrual cycles and were amenorrheic, respectively. Seventy-three (69.5%), 23 (21.9%), and 9 (8.6%) reported that their game performance was the best at the follicular phase, menses, and luteal phase of the menstrual cycle, respectively. No associations between the four reproductive-related variables were found. Postmenarche-trained players had significant lower age at menarche and higher percentage body fat. The somatotype profile registered lower ectomorphy rate for the postmenarche-trained participants after controlling for covariates. Early menarche group presented higher sum of six skinfold thickness and endomorphy rate compared to normal and late menarche groups. No differences were found when menstrual periodicity groups and best performance groups were compared, except for higher femur width in the regular menstrual cycle group compared to the irregular one. The association between body weight satisfaction and the four reproductive-related variables were not observed. Premenarche-trained Brazilian elite futsal players had the menarche later than the postmenarche-trained athletes. Most of the participants had menarche age classified as “normal,” presented “regular” menstrual cycles and perceived to perform better during the follicular phase of the menstrual cycle.

## Introduction

Exercise training can directly impact female reproductive health. Over 40 years of research have shown that female athletes are susceptible to oligomenorrhea and amenorrhea when compared to non-athletic populations (Dale et al., 1979; Frisch, 1980; Calthorpe et al., 2019). Moreover, a recent systematic review with meta-analysis revealed that age at menarche is 1.13 (95% CI: 0.80–1.47) years later in trained vs. non-trained girls (Calthorpe et al., 2019). It is worth mentioning that none of the included studies had team sport female athletes involved (Calthorpe et al., 2019).

Irregular menstrual cycle may pose as a risk for female reproductive health (Ackerman and Misra, 2018). However, later menarche can be protective against some chronic diseases when compared to having an early menarche. Epidemiological research has suggested that early age at menarche is associated with increased risk of cancer (Werneck et al., 2018a), higher blood pressure (Werneck et al., 2018b), gestational diabetes mellitus (Schoenaker and Mishra, 2017), and all-cause mortality (Tamakoshi et al., 2011). With regards to exercise performance, there is evidence that later menarche is associated with superior athletic performance (Stager et al., 1984), although the field of alterations related to the menstrual cycle and athletic performance has considerable controversial findings (e.g., no impact or some impact being previously reported; McNulty et al., 2020).

One factor that seems to influence later menarche is the onset of exercise training. Menstrual disturbances have been reported to be relatively more common in athletes who began training before the menarche. The premenarche-trained athletes reported a higher incidence of menstrual cycle irregularities than the athletes who began training after the menarche (Toriola, 1988). Frisch (1980) found that each year of training before menarche has accounted for 5 months of delay in the menarche in college athletes. Furthermore, premenarche-trained athletes presented high rates of irregular menstrual cycle (61%) and amenorrhea (22%; Frisch, 1980).

Menstrual dysfunctions can be caused by low energy availability or energy deficiency (Mountjoy et al., 2014; Ackerman and Misra, 2018). Low energy availability is linked with a disbalance between energy intake and energy expenditure, which can ultimately lead to lower body fat content (Loucks, 2004). Thus, low body fat, as a result of a disbalanced energy storage, can lead to changes in the menstrual cycle (Mountjoy et al., 2014) and may be a common characteristic of women with menstrual dysfunctions (Carlberg et al., 1983). Psychological aspects, such as eating disorder behaviors, can also influence female reproductive health by altering body weight and body fat content. Rates of eating disorders and body image alterations are 13.3 and 24.4% in female team sport athletes including futsal players (Kravchychyn et al., 2013).

The association between physical training and reproductive health issues, particularly in elite athletes, is a big concern of the sport and exercise scientific community. With regards to elite futsal players, to the best of our knowledge, the rates of menstrual dysfunctions have yet to be described. In fact, there is a lack of reproductive health-related information available in the team sport literature (Findlay et al., 2020). One potential explanation for a reduced focus on team sports is that research tends to focus on sports, which encourage leanness (Bruinvels et al., 2017), assuming that lower body fat and an ectomorphic somatotype would be potentially linked with higher rates of menstrual dysfunction (Mountjoy et al., 2014). To further explore this assumption, the present study aimed to characterize the age of onset of training, age at menarche, menstrual periodicity, and performance perception during the menstrual cycle and examined the impact of these reproductive variables on body composition, morphology, and body weight satisfaction in Brazilian elite futsal players. We hypothesized that premenarche-trained, late menarche, and athletes with irregular menstrual cycles would have greater rates of menstrual dysfunction, lower body fat, higher ectomorphy morphological profile, and greater dissatisfaction with body weight. We also hypothesized the study outcomes would not differ between menstrual periods when participants perceive to perform best.

## Materials and Methods

### Study Design and Subjects

Study design is cross-sectional. One-hundred and fifteen female Brazilian elite futsal players from the top national teams were recruited for the present study. The recruitment process was conducted through the Brazilian Confederation of Futsal with the support of the coaches of the teams. Eligibility criteria were checked and validated by the leading research investigator. The inclusion criterion was being registered for the twentieth Women’s Brazil Futsal Cup. No exclusion criteria were applied. The sample comprehended the population of female Brazilian elite futsal players. Since 1992, this national competition is a yearly event organized by the Brazilian Futsal Confederation^1^ between the 10 top teams in the country. One represents the host city and the other nine are champions from other Brazilian states. The study was approved by the Local Ethics Board (Process n. 039/2011) and complies with the Resolution of the National Health Council of the Brazilian Ministry of Health and the Declaration of Helsinki on human research. All players were fully informed about the purpose of the study and signed the consent form before any procedure took place.

### Measures

All measurements were conducted by the leading research investigator. The twentieth Women’s Brazil Futsal Cup format allowed each team to take 1 day off. In this way, data collection was performed in a room, in the same place, where the games were played and coincided with this day off of the teams, which allowed a minimum interval of 15 h between the last game and the evaluation. Thus, all the assessments were performed in a single day. Before performing the anthropometrical measurements, participants were interviewed following a structure interview with close-ended question and self-reported four reproductive-related variables:

1. Age of onset of training (years). This was measured based on a close-ended question (*what was your age, in years, of onset of training?*). Depending on the age at menarche, athletes were classified as premenarche-trained and postmenarche-trained (Frisch, 1980);
2. Age at menarche (years). This was measured based on a close-ended question (*what was your age, in years, at menarche?*). This variable was classified as early menarche (<12 years), normal menarche (12–14 years), and late menarche (>14 years; Day et al., 2015);
3. Menstrual periodicity. This was measured based on a close-ended question (for the most part of your reproductive years, what is your menstrual cycle periodicity? Options: 10–13 cycles/year; 3–9 cycles/year; and 0–2 cycles/year). Regular menstrual cycles were considered between 10 and 13 cycles/year, irregular menstrual cycles between 3 and 9 cycles/year and amenorrheic 0–2 cycles/year (Frisch, 1980);
4. Menstrual period when they performance best. This variable was obtained based on a close-ended question (*based on your perception, which menstrual period do you perform the best? Options: Menses; Luteal Phase; and Follicular Phase*). Based on their own perception, they answered one of the three options.

Besides that, they were also asked:

5. Ideal body weight (kg) and body weight satisfaction (classified as “Satisfied,” “No, increase,” and “No, decrease”). Ideal body weight was asked as a close-ended question (*what is your ideal body weight in kg?*). Body weight satisfaction was also a close-ended question (*are you satisfied with your body weight? Options: Yes; No, increase; and No, decrease*).
6. Information related to their training (i.e., volume and frequency). These data were also obtained with a close-ended question (*what is your training volume?* and *what is your training frequency?*). They were asked to report training volume in hours/week, minutes/week, or hours:minutes/week. We converted all answers in minutes/week before moving to data analysis. Training frequency was reported in times/week.

Anthropometric variables were checked in duplicate in the right hemisphere of the body. If the difference was greater than 0.2 mm for skinfolds or 0.5 cm for other variables, a third measurement was performed. The final result used for data analysis was the average of two or three measurements. All anthropometric measurements were performed with athletes wearing no shoes and only light clothing, in accordance with standardized procedures (Marfell-Jones et al., 2006). They have been already described in more details elsewhere (Queiroga et al., 2019). Measurements were taken in a private room at approximately the same time of the day for all participants during a period of 5 days. Body mass was assessed by a 100-g precision anthropometric scale (Welmy™ São Paulo, Brazil) and height was measured by a 0.1-cm precision wall-mounted stadiometer. Body mass index (BMI) was calculated as body mass (kg)/height squared (m^2^). Biceps girth (mid-upper-arm) and calf girth (cm) were measured using a non-elastic tape to the nearest 0.1 cm (Mabis™ Curitiba, Brazil). The Biceps girth (cm) was obtained with the arm in a tensed position, while calf girth (cm) was measured in a seated position with legs on the ground. Biepicondylar humerus and femur width were measured to the nearest 0.1 cm with a metal caliper (Somet™ Curitiba, Brazil).

Skinfold thickness (SKFT) was measured at six sites (triceps, subscapular, supraspinal, suprailiac, midthigh, and medial calf) to the nearest 0.1 mm with a Cescorf caliper (Cescorf™ Porto Alegre, Brazil).

The sum of three skinfold thicknesses (3SKFT; triceps + suprailiac + midthigh) determined body density (Jackson et al., 1980). Body fat percentage (%BF) was subsequently estimated (Siri, 1961). Fat mass (FM) and lean body mass (LBM) were calculated in kg: FM = (%BF/100) × body mass; LBM = body mass − FM. The three somatotype components (i.e., endomorphy, mesomorphy, and ectomorphy) were determined according to the Heath and Carter Anthropometric Somatotyping Method (Carter and Health, 1990). All variables were measured in duplicate in the right side of the body. A third measure was taken if a difference greater than 0.2 mm for skinfold thickness or 0.5 cm difference for all the other variables was recorded. The final score used in the data analysis was the mean of the two scores or the median of three scores. The technical error of measurement of the leading research investigator who conducted all the measurements was between 2.9 and 3.5% for SKFT and between 0.1 and 1.8% for the other anthropometric measurements. The intra-class correlation for the measurements varied between 0.98 and 0.99 for SKFT and between 0.95 and 0.99 for the other measurements.

### Statistical Analysis

Data were descriptively analyzed using mean ± standard deviation (SD), frequencies, and percentages. The associations between the four reproductive-related variables and body weight satisfaction were tested with Chi-square test. When more than 20% of cells had expected count less than 5, the Likelihood Ratio correction was applied. Cramer’s V was calculated as an effect size measurement of the associations.

Body composition and morphological variables were compared according to the four reproductive-related variables with one-way ANCOVA adjusting for the time of experience in the sport, training volume, and frequency. Effect sizes for comparisons were computed using partial eta squared (*η*^2^). Bonferroni correction was applied when significant differences were captured in the adjusted one-way ANCOVA. Significance was set at *p* < 0.05 for all analyses.

## Results

One-hundred and fifteen female Brazilian elite futsal players (age: 22.0 ± 3.9 years; body weight: 58.6 ± 7.6 kg; height: 161.8 ± 6.5 cm; BMI: 22.3 ± 2.2 kg/m^2^; and body fat: 22.2 ± 5.0%) were evaluated; however, four did not report the age at menarche. The average of their age at menarche was 13.1 ± 1.8 years, the time of experience was 9.0 ± 4.3 years, the training volume was 171.9 ± 77.4 min/week, and the training frequency was 5.2 ± 1.8 times/week.

Fifty-nine (53.2%) players were postmenarche-trained and 52 (46.8%) were premenarche-trained. Eighteen (16.2%), 65 (58.6%), and 28 (25.2%) were classified as early, normal, and late menarche, respectively. Only 11 (9.6%) and 1 (0.9%) had irregular menstrual cycles and were amenorrheic, respectively. Seventy-three (69.5%), 23 (21.9%), and 9 (8.6%) reported that their game performance was the best at the follicular phase, menses, and luteal phase of the menstrual cycle, respectively. No associations between the four reproductive-related variables were found (Table 1).

**Table 1:** Associations between the four reproductive-related variables.

Onset of training	Age of menarche			*p*	Cramer’s V
	Early	Normal	Late		
Premenarche-trained	6	29	17	0.164	0.180
Postmenarche-trained	12	36	11		
**Menstrual periodicity**					
Normal	16	58	25		
Irregular	2	6	3	0.887^*^	0.059
Amenorrheic	0	1	0		
**Best performance**					
Menses	4	13	4		
Follicular phase	12	42	19	0.869^*^	0.076
Luteal phase	2	5	1		
**Menstrual periodicity**					
**Onset of training**	**Normal**	**Irregular**	**Amenor**		
Premenarche-trained	48	3	1	0.183^*^	0.163
Postmenarche-trained	51	8	0		
**Best performance**					
Menses	20	3	0		
Follicular phase	66	6	1	0.681^*^	0.104
Luteal phase	7	2	0		
**Best performance**					
**Onset of training**	**Menses**	**Follicular**	**Luteal**		
Premenarche-trained	10	31	6	0.204^*^	0.174
Postmenarche-trained	11	42	2		

Values of *p* represent Pearson Chi-Square test.

* Values of *p* represent Likelihood Ratio correction. Amenor. = Amenorrheic.

After adjusting for the time of experience in the sport, training volume, and frequency, postmenarche-trained players had significant higher age and lower age at menarche than premenarche-trained players. They also presented higher body fat (kg). The somatotype profile registered lower ectomorphy rate for the postmenarche-trained participants (Table 2).

**Table 2:** Age, training, and anthropometric characteristics of premenarche-trained and postmenarche-trained female Brazilian elite futsal players.

	Onset of training		*p*	*η*^2^
	Premenarche-trained (*n* = 52)	Postmenarche-trained (*n* = 59)		
Age (years)	20.9 ± 3.6	22.8 ± 4.0	**<0.001**	0.464
Age at menarche (years)	13.5 ± 1.6	12.7 ± 1.9	**0.024**	0.047
Training volume (min/week)	169 ± 64	174 ± 88	0.155	0.019
Training frequency (times/week)	5.6 ± 2.0	4.9 ± 1.6	0.231	0.013
Body weight (kg)	58.0 ± 5.9	59.5 ± 8.7	0.131	0.021
Height (cm)	163 ± 6	162 ± 7	0.825	<0.001
BMI (kg/m^2^)	21.9 ± 2.1	22.7 ± 2.2	0.057	0.034
6SKFT (mm)	94 ± 20	103 ± 27	0.054	0.035
Body fat (%)	21.0 ± 4.4	23.2 ± 5.4	0.054	0.035
Body fat (kg)	12.3 ± 3.5	14.1 ± 5.2	**0.040**	0.039
Lean mass (kg)	45.7 ± 3.9	45.4 ± 4.9	0.637	0.002
Humerus width (cm)	6.1 ± 0.3	6.1 ± 0.3	0.835	<0.001
Femur width (cm)	8.8 ± 0.4	8.9 ± 0.5	0.846	<0.001
Biceps girth (tensed) (cm)	25.8 ± 1.8	25.9 ± 1.8	0.406	0.007
Calf girth (cm)	33.8 ± 2.0	34.3 ± 2.0	0.228	0.014
Endomorphy	4.2 ± 1.0	4.7 ± 1.2	0.060	0.033
Mesomorphy	4.0 ± 1.0	4.3 ± 0.8	0.293	0.010
Ectomorphy	2.3 ± 1.1	1.9 ± 0.9	**0.032**	0.042

Data are presented as mean ± standard deviation. Bold represents statistically significant differences. Comparisons were performed with one-way ANCOVA adjusting for time of experience in the sport, training volume, and frequency. Min, minutes; BMI, body mass index; 6SKFT, sum of six skinfold thickness.

When women with early, normal, and late menarche were compared, applying the same adjustments, we observed higher of sum of the six SKFT and endomorphy rate for the early menarche group compared to both normal and late menarche groups (Table 3). No significant results were evidenced in the comparison between women with regular vs. irregular menstrual cycle, except for higher femur width in the regular menstrual cycle group (Table 4). Women who perceived their performance to be the best at menses compared to follicular phase or luteal phase also did not differ for any anthropometric variable (Table 5).

**Table 3:** Age, training, and anthropometric characteristics of female Brazilian elite futsal players classified as early, normal, and late menarche.

	Age of menarche			*p*	*η*^2^
	Early (*n* = 18)	Normal (*n* = 65)	Late (*n* = 28)		
Age (years)	21.6 ± 4.0	21.9 ± 4.1	22.1 ± 3.4	0.853	0.003
Age at menarche (years)	10.3 ± 1.2	12.9 ± 0.7^†^	15.4 ± 0.7^†,‡^	**<0.001**	0.795
Training volume (min/week)	188 ± 103	164 ± 71	179 ± 74	0.246	0.026
Training frequency (times/week)	4.7 ± 1.8	5.3 ± 1.6	5.3 ± 2.1	0.365	0.019
Body weight (kg)	59.1 ± 9.9	58.6 ± 7.3	59.3 ± 6.6	0.811	0.004
Height (cm)	163 ± 7	162 ± 6	162 ± 7	0.789	0.004
BMI (kg/m^2^)	22.2 ± 2.9	22.3 ± 2.0	22.5 ± 2.1	0.873	0.003
6SKFT (mm)	113 ± 32	97 ± 23^†^	94 ± 20^†^	**0.013**	0.080
Body fat (%)	24.7 ± 6.1	21.7 ± 4.8	21.6 ± 4.5	0.050	0.056
Body fat (kg)	15.0 ± 5.8	12.9 ± 4.5	13.0 ± 3.8	0.160	0.034
Lean mass (kg)	44.1 ± 5.6	45.6 ± 4.2	46.3 ± 4.1	0.315	0.022
Humerus width (cm)	6.1 ± 0.3	6.0 ± 0.3	6.1 ± 0.3	0.285	0.024
Femur width (cm)	8.9 ± 0.6	8.8 ± 0.5	8.8 ± 0.4	0.705	0.007
Biceps girth (tensed) (cm)	26.0 ± 2.4	25.7 ± 1.6	26.3 ± 1.8	0.252	0.026
Calf girth (cm)	33.6 ± 2.5	34.1 ± 1.9	34.1 ± 1.7	0.632	0.009
Endomorphy	5.1 ± 1.5	4.4 ± 1.0^†^	4.3 ± 1.0^†^	**0.030**	0.065
Mesomorphy	4.1 ± 1.1	4.1 ± 0.8	4.2 ± 1.0	0.815	0.004
Ectomorphy	2.2 ± 1.2	2.0 ± 0.9	2.0 ± 1.0	0.821	0.004

Data are presented as mean ± standard deviation. Bold represents statistically significant differences. Comparisons were performed with one-way ANCOVA adjusting for time of experience in the sport, training volume, and frequency. ^†^Statistically different from Early group. Min, minutes; BMI, body mass index; 6SKFT, sum of six skinfold thickness. ‡Statistically different from Normal group.

**Table 4:** Age, training, and anthropometric characteristics of female Brazilian elite futsal players classified as regular and irregular menstrual cycle.

	Menstrual periodicity			*p*	*η*^2^
	Regular (*n* = 103)	Irregular (*n* = 11)	Amenorrheic (*n* = 1)		
Age (years)	22.0 ± 3.8	22.0 ± 4.2	---	0.166	0.032
Age at menarche (years)	13.2 ± 1.7	12.5 ± 2.5	---	0.606	0.009
Training volume (min/week)	176 ± 82	164 ± 71	---	0.748	0.005
Training frequency (times/week)	5.2 ± 1.8	5.1 ± 1.6	---	0.974	<0.001
Body weight (kg)	59.0 ± 7.8	55.5 ± 5.1	---	0.302	0.022
Height (cm)	162 ± 7	158 ± 5	---	0.199	0.029
BMI (kg/m^2^)	22.4 ± 2.2	22.2 ± 2.0	---	0.713	0.006
6SKFT (mm)	99 ± 25	98 ± 28	---	0.636	0.008
Body fat (%)	22.3 ± 5.2	21.5 ± 5.0	---	0.636	0.008
Body fat (kg)	13.4 ± 4.7	12.1 ± 3.7	---	0.468	0.014
Lean mass (kg)	45.6 ± 4.6	43.4 ± 2.9	---	0.373	0.018
Humerus width (cm)	6.1 ± 0.3	6.0 ± 0.2	---	0.877	0.002
Femur width (cm)	8.9 ± 0.5	8.5 ± 0.4	---	**0.037**	0.059
Biceps girth (tensed) (cm)	25.9 ± 1.9	25.4 ± 1.4	---	0.779	0.005
Calf girth (cm)	34.1 ± 2.0	33.3 ± 1.9	---	0.394	0.017
Endomorphy	4.5 ± 1.1	4.6 ± 1.3	---	0.840	0.003
Mesomorphy	4.1 ± 0.9	4.2 ± 1.0	---	0.999	<0.001
Ectomorphy	2.1 ± 1.0	1.9 ± 1.0	---	0.756	0.005

Data are presented as mean ± standard deviation. Bold represents statistically significant differences. Comparisons were performed with one-way ANCOVA adjusting for time of experience in the sport, training volume, and frequency. Min, minutes; BMI, body mass index; 6SKFT, sum of six skinfold thickness.

**Table 5:** Age, training, and anthropometric characteristics of female Brazilian elite futsal players according to the menstrual phase that they perceive to perform best.

	Best performance			*p*	*η*^2^
	Menses (*n* = 23)	Follicular (*n* = 73)	Luteal (*n* = 9)		
Age (years)	23.3 ± 3.3	21.5 ± 4.0	21.0 ± 4.6	0.519	0.013
Age at menarche (years)	12.7 ± 2.0	13.2 ± 1.8	12.3 ± 2.1	0.234	0.030
Training volume (min/week)	175 ± 82	173 ± 81	193 ± 84	0.768	0.005
Training frequency (times/week)	4.8 ± 2.4	5.2 ± 1.7	5.3 ± 0.5	0.188	0.033
Body weight (kg)	58.3 ± 7.9	58.7 ± 7.6	61.6 ± 7.8	0.464	0.015
Height (cm)	163 ± 7	162 ± 6	163 ± 6	0.915	0.002
BMI (kg/m^2^)	22.0 ± 2.1	22.4 ± 2.2	23.3 ± 2.4	0.315	0.023
6SKFT (mm)	100 ± 24	101 ± 26	96 ± 29	0.923	0.002
Body fat (%)	22.2 ± 4.8	22.8 ± 5.2	21.6 ± 5.7	0.898	0.002
Body fat (kg)	13.1 ± 4.1	13.6 ± 4.8	13.6 ± 5.1	0.955	0.001
Lean mass (kg)	45.1 ± 5.3	45.1 ± 4.3	48.0 ± 3.9	0.176	0.034
Humerus width (cm)	6.0 ± 0.3	6.1 ± 0.3	6.1 ± 0.3	0.753	0.006
Femur width (cm)	8.8 ± 0.4	8.9 ± 0.5	8.9 ± 0.6	0.432	0.017
Biceps girth (tensed) (cm)	25.6 ± 1.5	25.8 ± 1.8	26.7 ± 2.4	0.251	0.028
Calf girth (cm)	33.3 ± 1.9	34.1 ± 1.9	34.8 ± 2.4	0.122	0.042
Endomorphy	4.6 ± 1.0	4.6 ± 1.2	4.5 ± 1.5	0.902	0.002
Mesomorphy	3.8 ± 0.8	4.2 ± 0.9	4.4 ± 1.1	0.169	0.035
Ectomorphy	2.2 ± 1.0	2.0 ± 1.0	1.7 ± 1.0	0.469	0.015

Data are presented as mean ± standard deviation. Comparisons were performed with one-way ANCOVA adjusting for time of experience in the sport, training volume, and frequency. Min, minutes; BMI, body mass index; 6SKFT, sum of six skinfold thickness.

After adjusting for time of experience in the sport, training volume, and frequency, the perception of ideal body weight and difference between ideal and real body weight were not different between categories of onset of training, age of menarche, menstrual periodicity, and menstrual period when athletes reported to perform best (Table 6).

**Table 6:** Perception of ideal body weight and difference between ideal and real body weight according to categories of onset of training, age of menarche, menstrual periodicity, and menstrual period when athletes reported to perform best.

Onset of training	Ideal BW (kg)	Diff ideal-real BW
Premenarche-trained (*n* = 50)	57.1 ± 4.7	−1.1 ± 3.7
Postmenarche-trained (*n* = 57)	57.7 ± 6.7	−2.0 ± 3.6
*p*	0.172	0.202
*η*^2^	0.018	0.016
**Age of menarche**		
Early (*n* = 17)	57.1 ± 6.7	−2.3 ± 4.9
Normal (*n* = 62)	57.4 ± 5.8	−1.4 ± 3.5
Late (*n* = 28)	57.7 ± 5.7	−1.5 ± 3.2
*p*	0.950	0.447
*η*^2^	0.001	0.016
**Menstrual periodicity**		
Regular (*n* = 99)	57.6 ± 6.1	−1.6 ± 3.7
Irregular (*n* = 11)	54.6 ± 2.6	−0.9 ± 3.6
Amenorrheic (*n* = 1)	---	---
*p*	0.342	0.426
*η*^2^	0.020	0.016
**Best performance**		
Menses (*n* = 21)	57.6 ± 6.6	−1.1 ± 3.5
Follicular phase (*n* = 71)	57.0 ± 5.7	−1.9 ± 3.8
Luteal phase (*n* = 9)	60.0 ± 5.5	−1.6 ± 3.5
*p*	0.383	0.888
*η*^2^	0.020	0.003

Data are presented as mean ± standard deviation. Comparisons were performed with one-way ANCOVA adjusting for time of experience in the sport, training volume, and frequency. BW, body weight.

**Figure 1:**
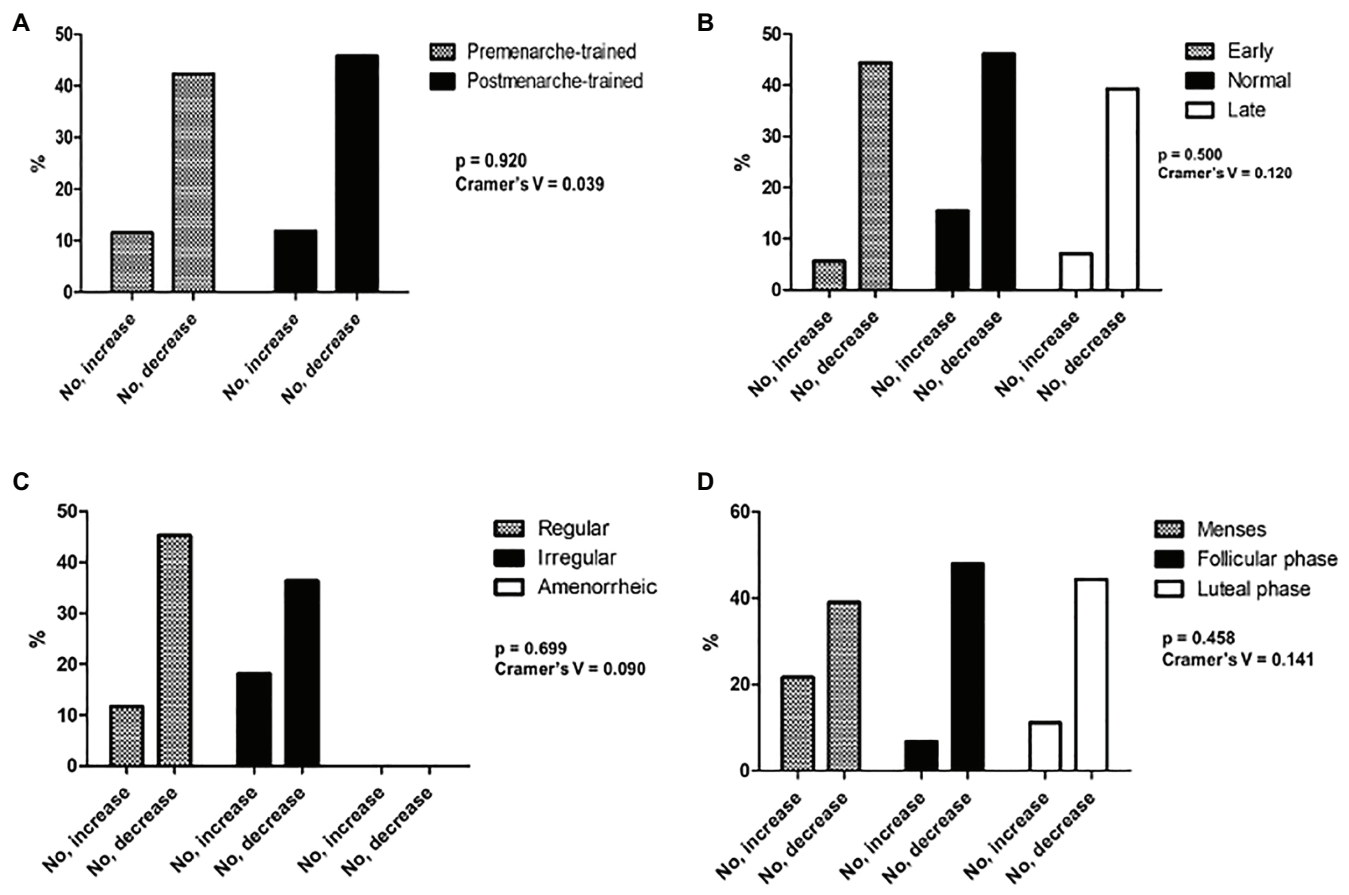
Association between body weight satisfaction and the four reproductive-related variables **(A–D)**.

The association between body weight satisfaction and the four reproductive-related variables is represented in Figures 1A–D. No significant results were found.

## Discussion

The purpose of this study was to characterize the age of onset of training, age at menarche, menstrual periodicity, and performance perception during the menstrual cycle and examined the impact of these reproductive variables on body composition, morphology, and body weight satisfaction in Brazilian elite futsal players.

The main findings of this research were that the number of female futsal players who engaged on the sport before menarche is balanced with the number of women who embarked on futsal training after the menarche. Those who engaged before the menarche had the menarche significantly later than those who started training after the menarche. Premenarche-trained players had lower body fat (kg) and greater ectomorphy profile after controlling for time of experience in the sport, training volume, and frequency. In addition, our descriptive analysis showed that most of the participants of the present study had their menarche age classified as “normal,” presented a “regular” menstrual cycle and perceived to perform better during the follicular phase of the menstrual cycle. Nonetheless, these factors did not associate with each other.

Intense training has been found to delay the onset of puberty in females by altering normal hormonal development (Theintz, 1994; Matina and Rogol, 2011; Malina et al., 2013). A systematic review with meta-analysis has shown that age at menarche is 1.13 (95% CI: 0.80–1.47) years later in trained vs. non-trained girls (Calthorpe et al., 2019). The age difference between premenarche-trained vs. postmenarche-trained in the present study is about 1.9 years.

Besides later menarche, female athletes are known to be more susceptible to oligomenorrhea and amenorrhea when compared to non-athletic populations (Dale et al., 1979; Frisch, 1980; Calthorpe et al., 2019). A relevant biological causal factor for the menstrual dysfunctions is low energy availability (Ackerman and Misra, 2018). Although we did not measure energy intake or energy balance, premenarche-trained futsal players had lower body fat (kg) than their postmenarche-trained counterparts. The ranges of body fat in our study are within the range from other studies with female futsal players of different performance levels (i.e., elite, sub-elite, and amateur; Barbero-Alvarez et al., 2015; Ramos-Campo et al., 2016; Beato et al., 2018; Palucci Vieira et al., 2020).

Also, our study demonstrated that women with early menarche presented higher sum of six SKFT and endomorphy rate compared to women whose menarche was classified as normal and late. The relationship between early age at menarche and higher adiposity is not completely elucidated; however, three factors have been suggested: (i) early menarche girls have longer exposure to a positive energy balance and (ii) endocrine factors, such as high levels of estrogen and GnRH, may accelerate pubertal development and increase subcutaneous body fat accumulation (Garn et al., 1986; Cole, 2003).

On the other hand, no differences in body fat were found in the comparison between groups related to menstrual periodicity (i.e., regular vs. irregular) and menstrual period when performance is perceived to be the best (i.e., menses, follicular, or luteal phases). Other anthropometric, body composition, and morphology variables did not differ between the four reproductive-related variables in Brazilian elite futsal players, except for femur width that was higher in the group with regular menstrual cycles compared to the irregular menstrual cycle group. Despite the significant results, this difference was only about 0.4 cm, which does not seem to be clinically important. Thus, our findings suggest that changes in reproductive variables are not related to relevant physical changes in this population. This agrees with Merzenich et al. (1993) who showed that exposure to exercise training may affect age at menarche through pathways that may go beyond adiposity.

Moreover, the percentage of women with normal age range menarche and regular menstrual cycle was much higher (58.6% for normal age range at menarche and 89.5% for regular menstrual cycle) in the present study compared to others. Frisch (1980) found that 61 and 22% of the premenarche-trained swimmers and runners had irregular menstrual cycle and were amenorrheic, and 60% of the postmenarche-trained swimmers and runners had regular menstrual cycles. In our study, 92.3% of the premenarche-trained and 86.4% of the postmenarche-trained had regular menstrual cycle. The reason for high rates of regular menstrual cycle can be related to the training volume of Brazilian female futsal players. Both groups had low training volume. Delayed age at the first menarche has been reported in young girls involved in athletic training for at least 15 h/week (Thomis et al., 2005). In the present study, the post-menarche-trained and pre-menarche-trained reported a much lower training volume, which was 174 ± 88 min and 164 ± 64 min (≈3 h/week – 2–6 h/week), respectively. Thus, the low training volume of the present sample is probably a strong contributor of the low rates of menstrual dysfunctions in Brazilian elite futsal players.

Another factor previously associated with menstrual dysfunctions is eating disorder behaviors (Mountjoy et al., 2014; Ackerman and Misra, 2018). A previous study with female athletes of team sports, including futsal, noticed that 13.3% of the sample had eating disorders (Kravchychyn et al., 2013). The authors also found that 24.4% of the sample presented body image distortion, and the team sport athletes of higher BMI and percentage body fat had greater risk for body image distortion (Kravchychyn et al., 2013). In the present study, we did not find associations between body weight satisfaction and the four reproductive-related variables. However, our data demonstrate large rates of body weight dissatisfaction, with most of the participants willing to decrease their body weight.

The majority of our sample perceived their performance to be the best in the follicular phase of the menstrual cycle and only a few participants said that the luteal phase was their best period for performing. This can be supported by physical and physiological data, indicating a reduction in maximal endurance performance during the luteal phase of the menstrual cycle in nine sub elite female soccer players (Julian et al., 2017). However, these authors did not find the same effect or jumping and sprint performance (Julian et al., 2017). Evidence suggests that maximum endurance performance during the menstrual cycle is at its lowest during menstruation and/or the luteal phase (Janse de Jonge, 2003). With regards to strength-related variables, a systematic review that investigated changes in strength-related variables during different phases of the menstrual cycle in eumenorrheic women recommends caution in the interpretation of results due to the methodological shortcomings identified by the quality assessment (Blagrove et al., 2020). Strength-related measures appear to be minimally altered (g ≤ 0.35) by the fluctuations in ovarian sex hormones that occur during the menstrual cycle (Blagrove et al., 2020). Given the aerobic predominance of futsal, it is expected that women would perceive the follicular phase as the best to perform. The physiological mechanism behind the explanation for a greater performance perception in the follicular phase can be related to the link between the follicular phase with reduced sympathetic autonomic activity and lower emotional stress (Tada et al., 2017). Recent research has suggested that greater parasympathetic activity is associated with increased performance in futsal players (Nakamura et al., 2020). Nonetheless, women who perceived their performance to be the best during the follicular phase compared to the other menstrual phases did not differ in the onset of training, anthropometric characteristics, and the perception of ideal body weight.

Our study has four important strengths. First, we characterized reproductive-related variables and menstrual dysfunctions in elite futsal players. In addition, we assessed women who participate in the largest futsal competition in Brazil, which allow us to have a representative sample of female Brazilian elite futsal players. The third strength is the strong and standard methodological procedures to determine anthropometric variables and, subsequently, body composition and morphological variables. The last point is the inclusion of relevant physical aspects (i.e., anthropometric variables) and a perceptual aspect (i.e., body weight satisfaction) that could be linked with menstrual dysfunctions. We also have limitations. Although we were able to access a large cohort of Brazilian elite futsal players, the lack of a longitudinal design (i.e., only a cross-sectional study design) represents an important limitation. Age at menarche and age at the onset of training are susceptible to recall bias. However, previous investigations have indicated that woman’s first menstrual bleed is a widely used measure of puberty timing, a distinct and notable event in their lives, and is well-recalled (Koo and Rohan, 1997; Bosetti et al., 2001; Must et al., 2002). We could not include more sophisticated measurements of body composition (i.e., DEXA scans and pletismography) due to the design of the study and the lack of time and resources to account for these measurements in real-world settings. Only doubly indirect measurements were included (i.e., anthropometry). Body weight satisfaction was not determined with validated questionnaires and is based on the instrument developed by the leading investigator for this study. Finally, other factors that might influence menstrual dysfunctions were not included in our analysis, such as socioeconomic conditions, nutritional assessment, and access to preventive health care, that may influence the timing and progression of puberty and reproductive health (Baxter-Jones and Maffulli, 2002; American Academy of Pediatrics Committee on Adolescence et al., 2006). Although not the ideal variable to represent socioeconomic conditions, we have found that 69.6% of our sample received a salary as a futsal player and receiving a salary was associated with being a premenarche-trained athlete and greater rates of late menarche (data not shown).

Considering the low effect size for almost all the analysis, we cannot provide general clinical/practical guidance and an anthropometric profile based on reproductive-related factors in female futsal players. Rather than that, monitoring these reproductive factors and accounting for their impact on futsal athletes should be done using an individualized approach and considering each athlete’s needs. Future research should longitudinally account for the impact of reproductive-related variables in performance and the body composition markers of female futsal players.

## Conclusion

Brazilian elite futsal players who engaged before the menarche had the menarche significantly later than those who started training after the menarche. Postmenarche-trained players had significant higher body fat and lower ectomorphy rate after controlling for covariates. Early menarche group presented higher sum of six skinfold thickness and endomorphy rate compared to normal and late menarche groups. No differences were found when menstrual periodicity groups and menstrual period, when performance is the best groups, were compared, except for higher femur width in the regular menstrual cycle group compared to the irregular one. Most of the participants of the present study had their menarche age classified as “normal,” presented a “regular” menstrual cycle and perceived to perform better during the follicular phase of the menstrual cycle. However, this perception was neither associated with other reproductive-related variables nor differed in anthropometric characteristics.

## Data Availability Statement

The datasets presented in this article are not readily available because of ethical reasons.

## Ethics Statement

The studies involving human participants were reviewed and approved by the Human Research Ethics Committee of the Midwest State University (UNICENTRO), process number 039/2011. The patients/participants provided their written informed consent to participate in this study.

## Author Contributions

MQ, DS, SF, MN, BP, MT, and EV: concept and design, methodology, the preparation of manuscript, the interpretation of data, formal analysis, and writing – review and editing. DS, TC, DF, and VW: formal analysis, the interpretation of data, writing and review, and the preparation of manuscript. MQ and SF: acquisition of subjects and data and writing and review. All authors contributed to the article and approved the submitted version.

### Conflict of Interest

The authors declare that the research was conducted in the absence of any commercial or financial relationships that could be construed as a potential conflict of interest.

## Footnotes

^1^http://www.cbfs.com.br
